# Novel method for detecting frequent TERT promoter hot spot mutations in bladder cancer samples

**DOI:** 10.1007/s10238-024-01464-3

**Published:** 2024-08-14

**Authors:** Ákos Kovács, Farkas Sükösd, Levente Kuthi, Imre M. Boros, Balázs Vedelek

**Affiliations:** 1 Department of Biochemistry and Molecular Biology, University of Szeged, Szeged, Hungary; 2 Department of Pathology, University of Szeged, Szeged, Hungary; 3 Hungarian Research Network Biological Research Center, Institute of Biochemistry, Szeged, Hungary; 4 Hungarian Research Network Biological Research Center, Institute of Genetics, Szeged, Hungary

**Keywords:** Bladder cancer, TERTp mutations, Novel method, Telomerase, Telomerase reverse transcriptase

## Abstract

Telomerase reverse transcriptase promoter (TERTp) mutations are frequently targeted tumor markers, however, they reside in regions with high GC content, which poses challenges when examined with simple molecular techniques or even with next-generation sequencing (NGS). In bladder cancer (BC), TERTp mutations are particularly frequent, however, none of the available tools have demonstrated efficacy in detecting TERTp mutations via a simple noninvasive technique. Therefore, we developed a novel PCR-based method for the detection of the two most common TERTp mutations and demonstrated its use for the analysis of BC samples. The developed SHARD-PCR TERTp mutation detection technique requires PCR and restriction digestion steps that are easily implementable even in less well-equipped laboratories. Cell lines with known mutational status were utilized for method development. Matching urine and tumor tissue samples from BC patients were analyzed, and the results were validated by next-generation sequencing. Analysis of eighteen urine and corresponding tumor tissue samples by SHARD-PCR revealed perfect matches in sample pairs, which paralleled the corresponding NGS results: fourteen samples exhibited mutations at the −124 position, two samples showed mutations at the −146 position, and no mutations were detected in two samples. Our study serves as a proof-of-concept and is limited by its small sample size, nonetheless, it demonstrates that SHARD-PCR is a simple, economic and highly reliable method for detecting TERTp mutations, which are common in different cancer types. For bladder cancer, SHARD-PCR can be performed with the use of noninvasive samples and could replace or complement currently used techniques.

**Supplementary Information:**

The online version contains supplementary material available at 10.1007/s10238-024-01464-3.

## Background

Telomeres are DNA‒protein complexes that protect linear chromosome ends [1]. Telomerase is inactive in most mature somatic cells, which causes these cells to undergo replicative senescence, limiting the number of division cycles that these cells can undergo [2]. In gametes, stem cells, intestinal crypts, hepatocytes, and most types of cancer cells, functional telomerase elongates telomeres, which provides virtual immortality to these cells [3].

In more than 85% of tumors of somatic origin, the transcriptionally silenced telomerase is reactivated [4]. Reactivation can occur for various reasons, frequently involving mutations in the telomerase reverse transcriptase promoter (TERTp) at positions 124 and 146 base pairs upstream of the translational starting site [4]. These alterations are sufficient to the formation of a de novo binding site for GABP, an E twenty-six (ETS) transcription factor. The binding of GABP to this site initiates euchromatin formation, ultimately activating TERT transcription [5, 6]. These mutations are particularly common in bladder cancer (BC), melanoma, and glioblastoma and hepatocellular carcinoma [7]. Since, TERTp mutation is a relatively early event in tumorigenesis [8–10], these nucleotide changes provide viable targets for diagnosis [11–13]. These markers also have prognostic value and are associated with decreased survival in papillary thyroid cancer patients and increased recurrence of urothelial BC [9, 14, and 15].

Detecting TERTp mutations by conventional PCR techniques is extremely challenging due to the high GC content (> 80%), the partially repetitive sequence and the structure of the promoter region [16]. Reliable yet resource-intensive methods, such as droplet digital polymerase chain reaction (ddPCR) [17–19] and gold-standard next-generation sequencing (NGS) [15, 20] methods, have been developed for detecting TERTp mutations. Nevertheless, even NGS has limitations in this region, resulting in low coverage [21, 22].

Among tumor types with a high frequency of TERTp mutations (68–69%) [4], bladder cancer is an appropriate target for noninvasive diagnosis given that tumor cells and cell-free tumor DNA appear in the urine and can be easily isolated and analyzed [20]. The current method for diagnosing BC involves the use of cystoscopy, a costly and unpleasant intervention. Typically, hematuria, a later occurring symptom, raises the possibility of BC and the necessity of cystoscopy, potentially leading to a delayed diagnosis. Consequently, there is a clear need for a cost-effective diagnostic and/or screening tool, as noninvasive tools developed in recent decades (e.g., antigen tests, ddPCRs, NGS methods) have not replaced cystoscopy. Moreover, the high cost makes the available techniques impractical for screening purposes.

Here, we present a new method that combines restriction digestion and PCR steps with specifically modified primers and adapters, making the detection of mutations readily achievable. In our study, we used both tumor and urine samples from BC patients and validated the results obtained by the use of the developed technique with NGS analysis of the same samples.

## Methods

### Cell lines and culture conditions

The methodology was refined using cell lines that had established mutational profiles according to the Cellosaurus database [23]. Specifically, the TERTp wild type HeLa cervical carcinoma, the -124 mutant Malme-3 M melanoma, and the -146 mutant UACC-257 melanoma cell lines were obtained from the American Type Culture Collection (ATCC). Cell lines were cultured in RPMI-1640 medium (Sigma-Aldrich), complemented with 10% fetal bovine serum, 2 mM glutamine and 1% penicillin–streptomycin. Cells were cultured at 37 °C under 5% CO_2_ and at 95% humidity.

### Clinical sample collection and storage

BC patient samples were provided by the Department of Pathology, University of Szeged. Urine samples were collected from patients before transurethral resection of bladder tumor (TURBT) or cystectomy between 2016 and 2020. The average age at the time of surgery was 64.8 years (20–85 years, median age 66 years). FFPE tissue samples were stored at 20 °C for 1–4 years, while frozen tissue samples were stored at − 80 °C for 1–4 years before processing. Each frozen tumor tissue sample was paired with frozen urine samples from the patient, collected before surgery, frozen in liquid nitrogen (2–3 h after sample collection) and stored at − 80 °C. FFPE samples were paired with fresh urine samples collected before surgery and processed the same day (3–8 h after sample collection). Altogether, 9 FFPE, 20 frozen tissue, 46 frozen urine and 15 fresh urine samples were processed. Some urine samples had no paired tissue samples, since the tumor size was too small, the whole sample was needed for histological assessment, or DNA extraction was unsuccessful, therefore, these urine samples were also excluded from our final 18-sample pair analysis.

### DNA preparation

For DNA isolation from cell cultures, we used “NucleoSpin Tissue Kit” (Macherey–Nagel) according to the manufacturer’s instructions.

Several distinct commercially available DNA isolation kits were used for the extraction of DNA from clinical samples. For DNA isolation from frozen tissue samples, we used “NucleoSpin Tissue Kit” (Macherey–Nagel) according to the standard protocol for tissue samples, as described in the user’s manual. For DNA sample preparation from FFPE samples, we used “QIAamp DNA FFPE Tissue Kit” (Qiagen), according to the user’s manual. Sample preparations from urine were performed using either “NucleoSpin Tissue Kit” (Macherey–Nagel) or “Quick-DNA™ Urine Kit” (Zymo Research). For “NucleoSpin Tissue Kit” (Macherey–Nagel), a 15 ml urine sample was centrifuged at room temperature for 10 min at 3000 × g. The sediment was resuspended in 1 ml of TE buffer (20 mM Tris, 5 mM EDTA, pH 8.0), loaded into a 1.5 ml microcentrifuge tube and centrifuged for 90 s at 11,000 × g. The supernatant was discarded, and further sample processing was carried out according to the user’s manual for cultured cells. For “Quick-DNA™ Urine Kit” (Zymo Research), sample processing was carried out according to the user’s manual.

To assess DNA concentration and purity NanoDrop-1000 spectrophotometer was used.

### Target amplification PCR

For amplification of the TERTp region, we used Q5 high-fidelity polymerase (New England Biolabs (NEB)) according to the following protocol. The 25 µl PCR mixture consisted of 5 µl of 5X Q5 Reaction Buffer (NEB), 5 µl of 5X Q5 High GC Enhancer (NEB), 489 Forward Primer and 489 Reverse Primer (Supplementary Table 1) at a final concentration of 0.4 µM, dNTP mix (0.2 mM each, Thermo Fisher Scientific), 5 mU Q5 High-Fidelity DNA Polymerase (NEB), 50–100 ng of template DNA and nuclease-free water. The PCR program started with primary denaturation at 98 °C for 5 min; 35 cycles of 98 °C for 30 s, 68 °C for 30 s, and 72 °C for 1 min; and a final elongation at 72 °C for 5 min.

All PCR programs were tested in various thermocyclers Veriti (Applied Biosystems), ProFlex (Applied Biosystems) and Arktik Thermo Cycler (Thermo Fisher Scientific).

### Processing of PCR products

PCR products were purified using the ethanol DNA precipitation method by the addition of 5X sample volume of 96% ethanol and 100 ng glycogen. The samples were incubated at − 20 °C for 1 h, after which the precipitated DNA was centrifuged at 11,000 × g for 10 min. The supernatant was carefully removed without disturbing the pellets. A 3 × sample volume of 70% alcohol was added, and the tubes were inverted several times. The samples were subsequently centrifuged at 11,000 × g for 5 min. The alcohol was removed, and the pellets were air-dried. The DNA was dissolved in 20 µl of TE buffer or nuclease-free water.

### Restriction digestion

Restriction digestion was performed by using HpyAV restriction endonuclease (NEB) for 2 h at 37 °C. The 20 µl digestion mixture consisted of 2 µl CutSmart Buffer (NEB), 0.4 µl HpyAV enzyme, 400 ng first PCR product and nuclease-free water.

### Selective high-sensitivity amplification of restricted DNA by PCR

The cleavage products of HpyAV digestion were amplified either with Q5 DNA polymerase and primers containing 3’ dideoxycytidine nucleotides or with DreamTaq DNA polymerase (Thermo Fisher Scientific) and primers with noncomplementary 3’ ends.

#### SHARD-PCR with Q5 polymerase

A total of 50–100 ng of digested DNA was used as a template in a 20 µl final volume PCR mixture, which consists of 4 µl of 5X Q5 Reaction Buffer (NEB) and 4 µl of 5X Q5 High GC Enhancer (NEB). The following primers were used: 0.5 nM Blocked Oligo A146-specific for − 146 M; 0.5 nM Blocked Oligo A124-specific for − 124 M; 5 nM Extension Primer 1; 5 nM Extension Primer 2; 1 µM Forward-Reverse Primer (Supplementary Table 1, dNTPs used in 0.05 mM each (Thermo Fisher Scientific); 4 mU Q5 High-Fidelity DNA Polymerase (NEB); and nuclease-free water. The PCR protocol included a primary denaturation step at 98 °C for 5 min; 30 cycles of 98 °C for 40 s and 77.5 °C for 30 s; 40 cycles of 98 °C for 40 s, 69 °C for 30 s, and 72 °C for 40 s; and a final elongation at 72 °C for 5 min.

#### SHARD-PCR with DreamTaq polymerase

The 20 µl PCR mixture consisted of 2 µl of 10X DreamTaq buffer (Thermo Fisher Scientific), primers used at a final concentration of 5 nM Blocked Oligo B146, specific for − 146 M; 5 nM Blocked Oligo B124, specific for − 124 M; 0.05 µM Extension Primer 1; 0.05 µM Extension Primer 2; 1 µM Forward-Reverse Primer (Supplementary Table 1), dNTPs used at 0.05 mM each (Thermo Fisher Scientific), 2.5 mU DreamTaq DNA Polymerase (Thermo Fisher Scientific), 50–100 ng of digested template DNA and nuclease-free water. The PCR protocol included primary denaturation at 98 °C for 5 min; 50 cycles of 98 °C for 1 min, 70 °C for 30 s, and 72 °C for 1 min; and a final elongation at 72 °C for 7 min.

### Gel electrophoresis and evaluation

The PCR products were analyzed via agarose gel electrophoresis (1.5–3% agarose gel, 1 × TAE buffer (40 mM Tris base (pH 8.3, not adjusted), 20 mM acetic acid, 1 mM EDTA) and visualized via ethidium bromide staining.

### NGS sample preparation and analysis

The TERTp region was amplified and elongated with an adaptor sequence compatible with the TruSeq Custom Amplicon Index Kit (96 indexes, Illumina) using the same PCR protocol as described in the “Target amplification PCR” section of the manual. Before indexing, the PCR products were purified via the ethanol–DNA precipitation method as described in the “Processing of PCR products” section. For indexing, the Illumina TruSeq Custom Amplicon library preparation protocol was followed. The templates were prepared by adding 20 µl of freshly diluted 50 mM NaOH to 1.5 µl of the resuspended DNA. The indexing PCR mixture consisted of 2 µl of i5 index primers, 2 µl of i7 primers, 10 µl of template, 10.78 µl of PMM2 solution, and 0.22 µl of TDP1 solution. The PCR protocol was as follows: primary denaturation at 95 °C for 3 min; 35 cycles of 95 °C for 30 s, 66 °C for 30 s, and 72 °C for 1 min; and a final elongation at 72 °C for 5 min. Clean-up was performed using AMPure XP beads (Beckman Coulter) according to the user’s manual. A quality check was performed using a Bioanalyzer with an Agilent DNA 1000 Kit according to the user’s manual. Indexed samples were pooled based on Agilent chip data into a 3.2 nM library. Sequencing was performed on an Illumina MiSeq instrument using a MiSeq Reagent Nano Kit V2 (300 cycles). Quality control and trimming were performed by FastQC and TrimGalore, respectively. The reads were aligned using the Bowtie algorithm to a 489 bp region of the human TERT promoter sequence (NC_000005.10[1294925..1295413]) as a template. Mutant and wild type reads were counted by Integrative Genomics Viewer software (IGV) [24].

## Results

### Sample collection and DNA isolation

**Fig. 1:**
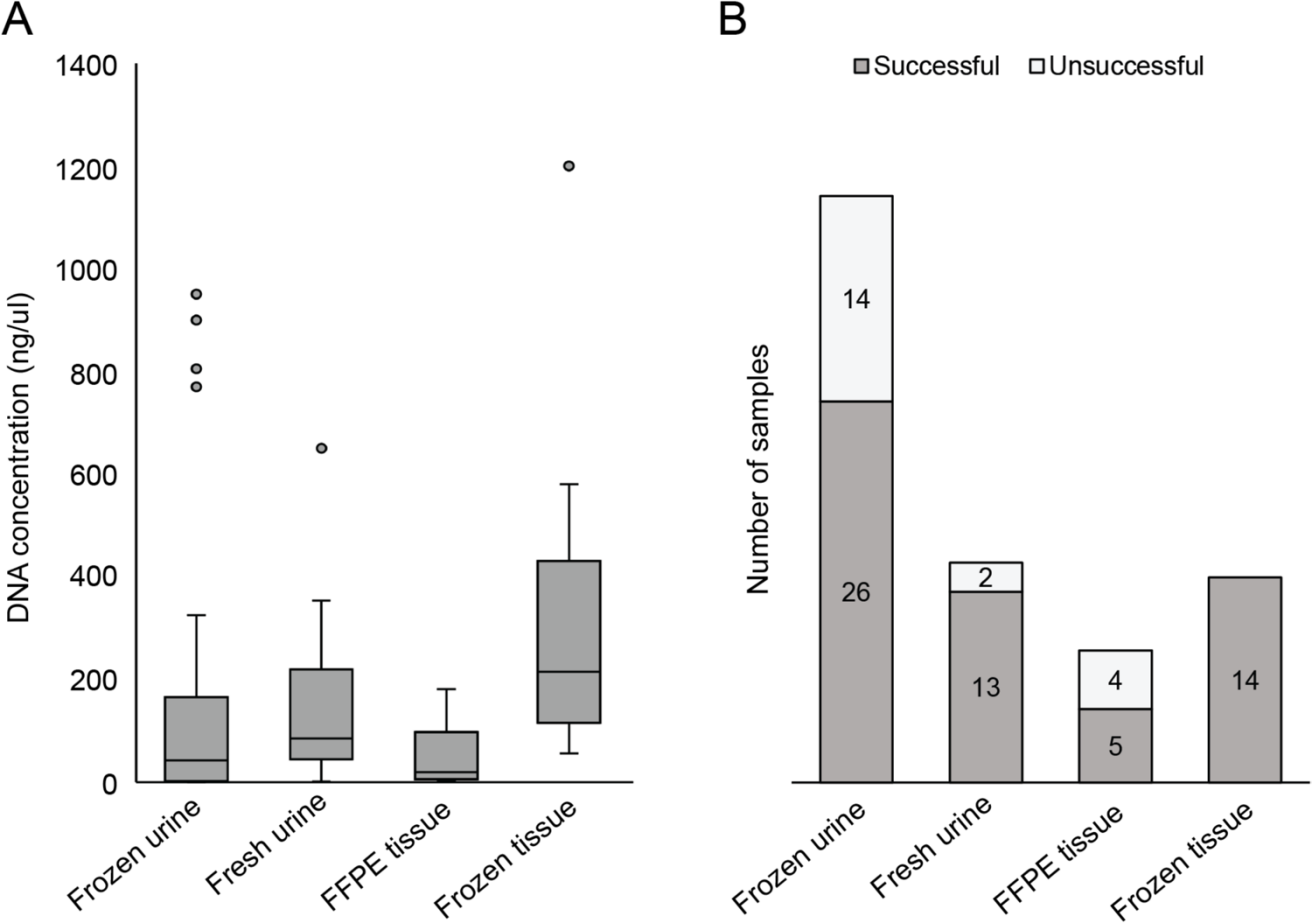
**A** DNA concentrations from different sample types **B** the success rate of the first PCR is strongly dependent on the DNA concentration. (FFPE tissue: formalin-fixed paraffin-embedded tissue)

DNA from the urine and tumor tissue samples of BC patients were examined in this study. Frozen tissue samples and fresh urine samples provided the best quality and yield of DNA (Fig. 1).

The daily first urine sample repeatedly resulted the highest yield of DNA, which is the most recommended sample for these analyses. DNA isolated from formalin-fixed paraffin-embedded (FFPE) tissue samples and frozen urine samples was generally more degraded, and the yield was also lower. FFPE tissue samples and frozen urine samples might be used, but the success rate of the first PCR step is much lower (Fig. 1).

### Target amplification PCR

The theoretical sensitivity of the target amplification PCR was assessed by the utilization of isolated promoter regions. Both the wild type and mutant promoter elements were amplified by PCR and inserted into the pJET1.2 cloning vector. Subsequently, the vectors were linearized and utilized in a dilution series ranging from 0.1 ng to 10^ − 5 ng as templates for the target amplification PCR (Supplementary Fig. 1). The lowest concentration at which a detectable PCR signal was observed was 0.454 pg of template, which corresponds to approximately 128,098 molecules. The mutant PCR fragments exhibited a stronger amplification than the wild type, but the difference was not significant. The performance of the PCR reaction was enhanced when utilizing genomic template, as a mere 50–100 ng of genomic DNA proved to be adequate for amplifying the target region. This observation is noteworthy as the genomic template theoretically contained fewer template molecules (15,480–30,960) compared to the 0.454 pg of plasmid [25]. However, such calculations should be considered as rough estimates as there could be significant differences in the mass and composition of the genomes of healthy and cancer cells. With lower amounts of genomic DNA, the success rate decreased. The DNA quality also influenced the results, as highly degraded genomic DNA samples (DNA fragments smaller than 489 bp) are not suitable for amplification in this PCR. Following target amplification, a clean-up step is necessary, since the PCR buffer is not suitable for restriction digestion. Furthermore, the clean-up step removes the residual primers from the first reaction, which otherwise might interfere with later steps of the protocol.

### Restriction digestion

The first two steps of the method are similar to those of the highly sensitive restriction fragment nested allele-specific PCR (RFN-AS-PCR) method, which was developed for the diagnosis of acute myeloid leukemia [26, 27]. In our approach, the aim of restriction digestion is not to improve specificity but rather to generate a DNA fragment that is unique to the mutation. At both hotspots of TERTp, the C- > T mutation creates a novel recognition site for the HpyAV restriction endonuclease (CCTTCN_6_). The 489 bp wild type target sequence that was amplified in the first PCR contained three HpyAV recognition sites, while the mutant sequence contained four. Despite, the relatively small fragments resulting from HpyAV cleavage, it is possible to differentiate the mutants and the wild type DNA from each other when the proportion of mutant alleles is high enough (Supplementary Fig. 2). In the tumor and urine samples, we expect that the proportion of mutant alleles is much lower due to sample heterogeneity. If the wild type DNA background is strong, signal amplification is needed for the detection of mutant alleles.

### PCR detection of the mutant DNA fragments

**Fig. 2:**
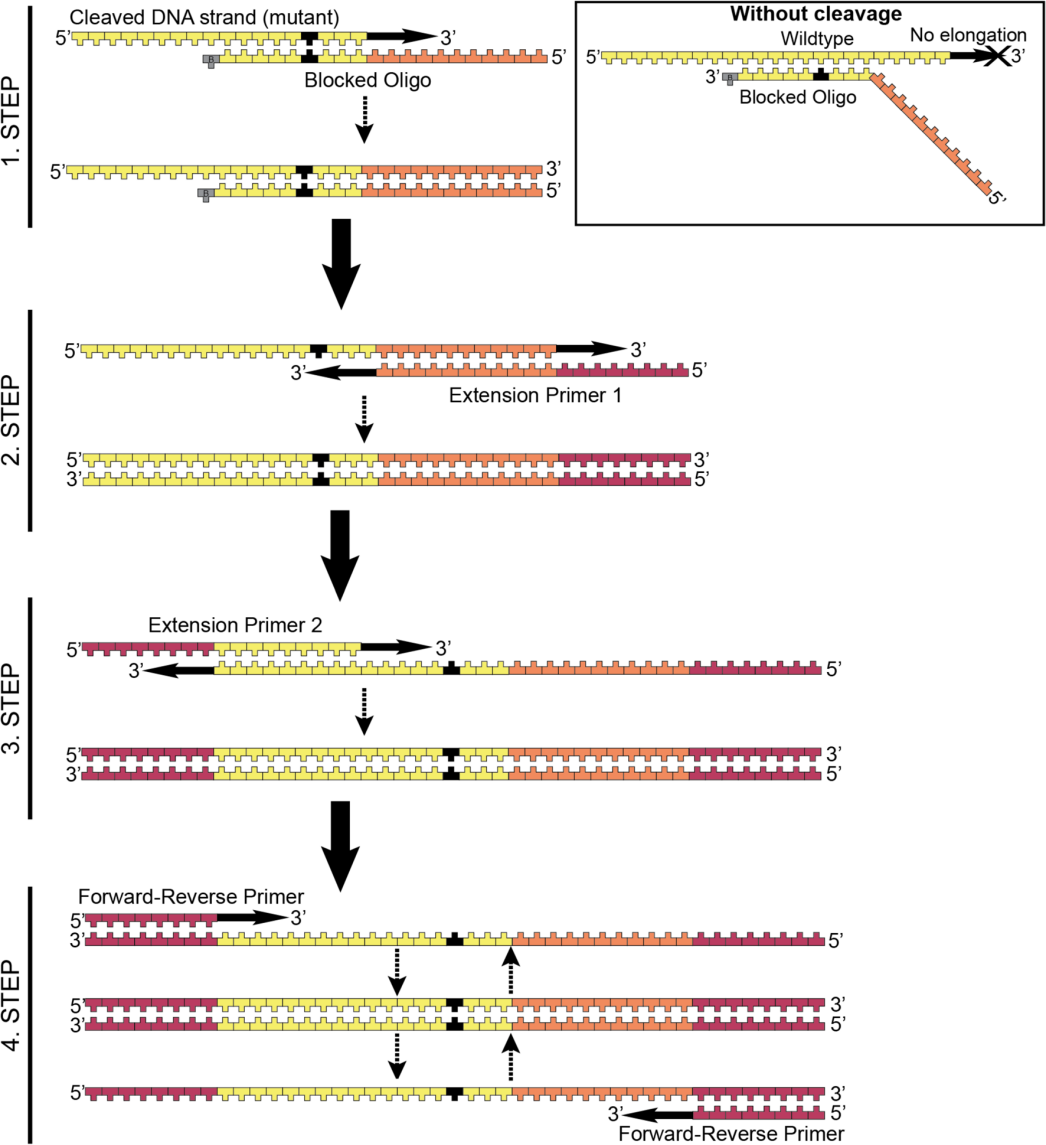
SHARD-PCR in detail: Step 1. The Blocked Oligo (3’ blocked with ddC or a noncomplementary end) binds to one strand of the cleaved DNA fragment specifically. This oligonucleotide is only partially complementary to the cleaved DNA fragment; the 5’ end is designed to overhang and serves as a template. The cleaved (original) DNA strand elongates using the 3’-blocked oligo’s overhanging 5’ end as a template. If the original DNA strand is wild type, it is not cleaved by the endonuclease, thus, elongation and subsequent steps do not occur. The product of this step is a single-stranded DNA fragment, with the original part (5’ end, yellow) and the “artificial” elongated part (3’ end, orange). Step 2. Extension Primer 1 binds to the elongated “artificial” DNA section of the last step’s product. Elongation occurs on both strands, since Extension Primer 1 is also designed to overhang (sequence added at the 3’ end, dark red). Here, the product is a double-stranded DNA fragment. Step 3. Extension Primer 2 binds to the new DNA strand, which is a product of Extension Primer 1. Elongation occurs on both strands, since Extension Primer 2 is also designed to have an overhang (with an added sequence on the 3’ end, dark red). As a result, a double-stranded DNA molecule with identical 3’ and 5’ ends is synthesized. With Extension Primer 1 and 2, a complete PCR could be performed. Step 4. Forward-Reverse Primer binds to identical extended DNA sections at the 5’ ends of both DNA strands, which allows a conventional PCR to occur

To overcome the wild type background problem, we designed an amplification protocol (Selective High-sensitivity Amplification of Restricted DNA by PCR or SHARD-PCR) to detect DNA fragments originating from the cleavage of the mutant promoter. First, a specific 3’ end-blocked adapter DNA was used, which consisted of two parts. The half from the 3’ end is complementary to the mutant DNA fragment, while the 5’ half is a unique DNA sequence designed by us. First, the 3’ end-blocked adapter DNA molecule hybridizes to both the wild type and the mutant DNA fragments. If the fragment is mutated, then it is cleaved, and therefore, it can be elongated, using the overhanging, single-stranded unique sequence of the adapter as a template. The wild type fragment, on the other hand, is unable to undergo elongation as a result of its extended 3’ end, which is not complementary to the unique 5’ sequence of the adapter. This step permits the selective extension of the cleaved mutant DNA, while the adapter used in this step does not allow the amplification of the target sequence, as its 3’ end is blocked. Two adapters for the two most common mutations can be used in the same reaction. Mutant DNA is selectively amplified with further primers, one complementary to the 5’ unique region of the adapter and the other to the DNA fragments generated by cleavage of the mutant DNA. This step also further elongates the target DNA to facilitate detection, and a 226 bp long PCR product for the − 124 mutant or a 205 bp long PCR product for the − 146 mutant is produced (Fig. 2).

It is crucial to adequately block primers because failure to do so may result in the generation of nonspecific signals. As the blocking reaction might not be satisfactory, we intended to lower the possibility of amplification of nonspecific products, thus, the primer mixture contained a low concentration of long “starter” primers (Blocked Oligo, and Extension Primer 1–2). However, for signal amplification, we used a shorter primer with a lower GC content (Forward-Reverse Primer). The Q5 version of this PCR included two different cycles. The first is optimized for the specific annealing of the starter primers at an exceptionally high temperature (77,5 °C) because of the high GC content. The second is optimized for signal amplification at a lower temperature (69 °C).

Two approaches were tested for SHARD-PCR. Q5 DNA polymerase combined with dideoxycytidine 3’ end-modified adapters was used in one approach, and DreamTaq DNA polymerase combined with adapters blocked with a noncomplementary 3’ end was used in the other approach. Both approaches were proven to be feasible (Supplementary Fig. 3), however, the cost-effectiveness of the DreamTaq enzyme and the noncomplementary primers were more favorable.

**Fig. 3:**
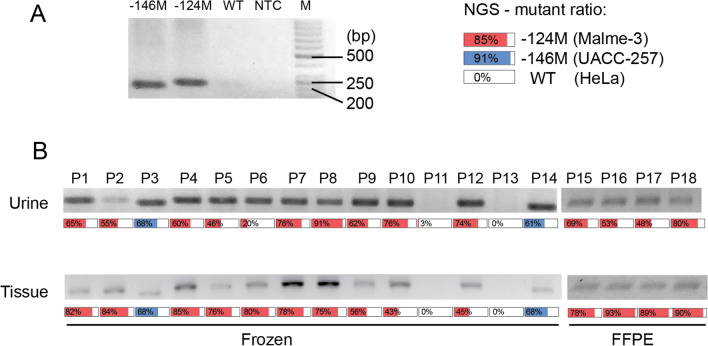
**A** Our method (SHARD-PCR) demonstrated its feasibility in the Malme-3 M, UACC-257 and HeLa cell lines. **B** Tumor-urine sample pairs from 18 patients were tested by our method and by NGS

During the development of the method, DNA obtained from the HeLa, Malme-3 M and UACC-257 cell lines was used. Malme-3 M and UACC-257 contained mutations of − 124 and − 146, respectively, while HeLa cells had the wild type TERT promoter (Fig. 3A).

Utilizing PCR products obtained from plasmid templates, we created a dilution series ranging from 50% mutant to 0.005% mutant promoter ratio by combining the wild type and mutant products. Subsequently, an analysis was conducted to determine the detectable mutant ratio using our methodology. It was observed that an estimated 0.5% of mutant ratio is detectable with confidence (Supplementary Fig. 1).

After successful trials with the cell lines, the method was tested on clinical samples. Eighteen urine-tumor sample pairs were included in the final analysis, and the results were validated by NGS. The corresponding tumor-urine samples presented identical mutations. Of the 18 samples, 14 had a promoter mutation at position − 124, two had a promoter mutation at position − 146, and in two samples no hotspot mutations were detected (Fig. 3B, Supplementary Table 2).

### NGS results

Sequencing of the TERTp amplicons yielded 76.45 Mbp, and 88.73% of the reads reached Q30. A total of 19.75% of the total reads were assigned to samples based on their index sequences. One sample contributed 0.395% of the total reads (median: 0.219%, min–max: 0.0148–2.26%). Indexed reads were aligned to the human TERTp region, which resulted in 242.3 average (median: 100) coverage at potential TERTp mutations. The unexpectedly low coverage despite sufficient input DNA is in accordance with the data in the literature, which reports difficulties in sequencing this genomic region [21, 22]. Both NGS and the present method verified the presence of the same mutations (Supplementary Table 2).

## Discussion

Various methodologies have been developed for the detection of TERTp mutations, and each approach comes with its own strengths and weaknesses.

Sanger sequencing offers dependable and high quality outcomes in determining short sequences and suitable for the detection of the TERTp alterations. Nevertheless, in order to accurately identify mutations, the proportion of mutant alleles in the sample should exceed 20%. As a result, the utility of Sanger sequencing in TERTp mutation detection is constrained to tissue samples, where the mutant DNA ratio is high, compared to urine samples, where the expected ratio of mutants is lower.

Several promising PCR based methods have been developed recently for the detection of TERTp mutation utilizing the amplification refractory mutation system (ARMS) or droplet digital polymerase chain reaction (ddPCR) techniques [18, 28–32]. These methods require relatively expensive machinery and fluorescent probes. They usually target one mutation per reaction, with high sensitivity and specificity. NGS techniques such as Illumina sequencing or Nanopore sequencing are also used to detect TERTp mutations [20, 21, and 33]. Despite, Illumina sequencing has low coverage on GC rich regions, the NGS techniques provide the most information of the mutation status of a promoter as not only the targeted mutations are detectable, but all changes in the sequence. Mass spectrometry approach can also detect the mutation without providing any sequence information [34]. Currently NGS and ddPCR are regarded as the gold standards in the field, characterized by their high sensitivity and specificity. However, these techniques and mass spectrometric approaches demand significant financial investment in equipment and a high level of technical expertise for both conducting the experiments and evaluating the results.

With the development of SHARD-PCR, our aim was to minimize resource requirements, including materials and machinery, and provide an affordable alternative for bladder cancer screening in less well-equipped laboratories. For the detection of the two most common TERTp mutations, our method consists of three major steps: target amplification PCR, mutation-specific restriction digestion and PCR amplification of cleaved mutant DNA fragments (SHARD-PCR). This method has proven to be feasible for the detection of mutations in extremely GC-rich regions.

Excluding the DNA isolation step, our SHARD-PCR technique proved to be approximately 20 times more economically efficient per sample than the NGS method we implemented. To determine the specificity and sensitivity of the methodology, more extensive research is needed, however, a promising aspect is the complete overlap observed between the NGS data and the data acquired using our approach. In addition, the target amplification PCR seems to preferentially amplify the mutant forms of the promoter, potentially due to its lower GC content (Supplementary Fig. 1). This could explain the higher-than-anticipated mutant ratio identified in our NGS outcomes. Based on the NGS data analysis, the mutant ratio in each of the mutant samples was found to be over 20%, significantly surpassing the detection threshold of SHARD-PCR. Despite, the need for further assessment regarding its clinical applicability, this method has potential for use in several tumor types characterized by a high TERTp mutation rate. Moreover, the fundamental principles of this approach can be extended to detect mutations beyond the scope of TERTp.

## Conclusions

We developed SHARD-PCR, a simple PCR-based method for detecting the two most common TERTp mutations in cancer. The adoption of this method could lead to significant clinical implications in the diagnosis and screening of bladder cancer.

## Supplementary Information

Below is the link to the electronic supplementary material.

Supplementary file1 (PDF 549 kb)

## Data Availability

The datasets used and/or analyzed during the current study are available from the corresponding author upon reasonable request.

## Abbreviations

BC: Bladder cancer
ddPCR: droplet digital polymerase chain reaction
FFPE: Formalin-fixed paraffin-embedded
NGS: Next-generation sequencing
PCR: Polymerase chain reaction
TERT: Telomerase reverse transcriptase
TERTp: Telomerase reverse transcriptase promoter
TURBT: Transurethral resection of bladder tumor
ARMS: Amplification refractory mutation system
