# Supplementary material for: Novel method for detecting frequent TERT promoter hot spot mutations in bladder cancer samples
Source: Clin Exp Med. 2024 Aug 14;24(1):192. doi: 10.1007/s10238-024-01464-3 (PMC11324672; doi:10.1007/s10238-024-01464-3)
Supplement: Supplementary file 1 — Supplementary file1 (PDF 549 kb) [file 10238_2024_1464_MOESM1_ESM.pdf]

Supplementary information

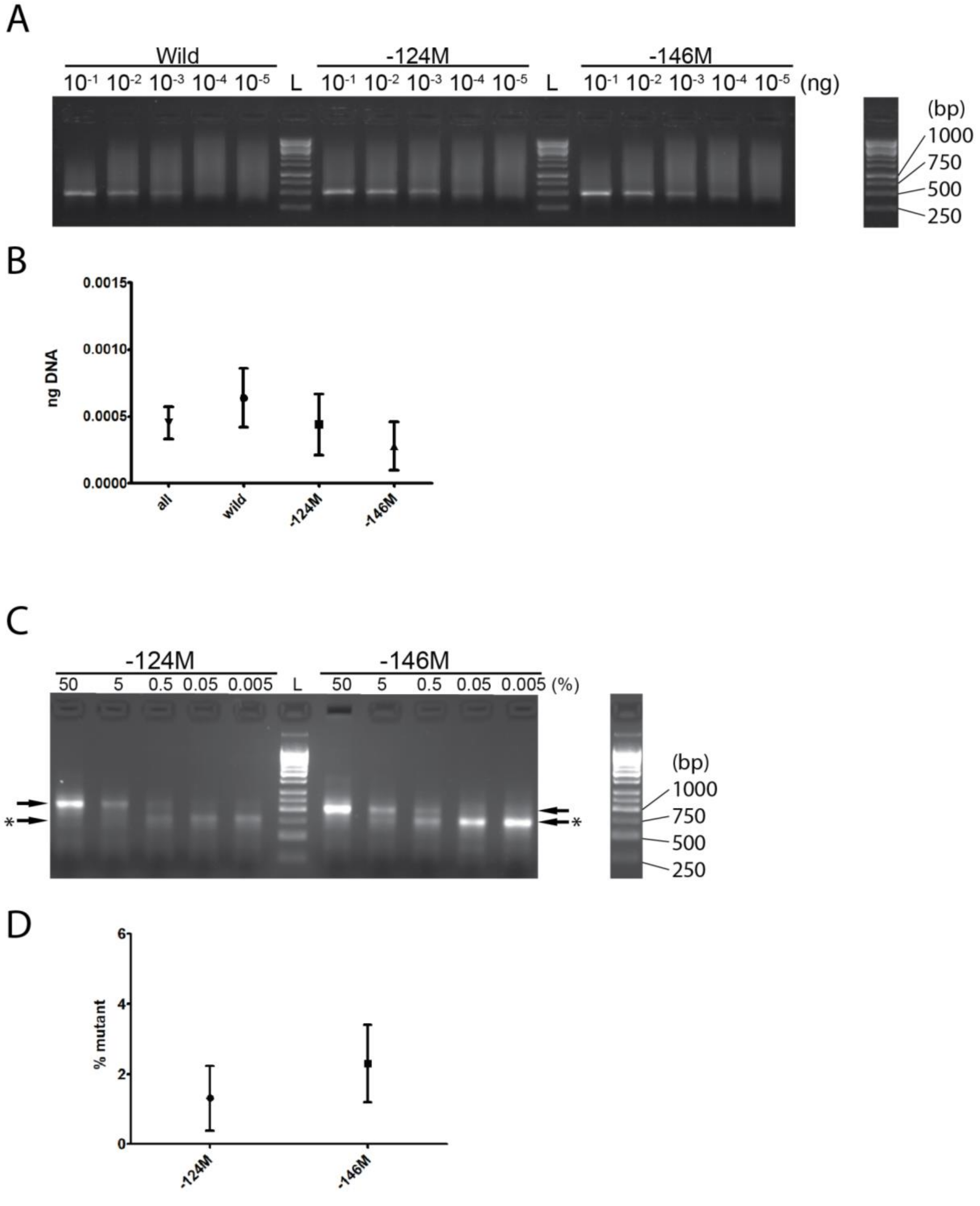

Supplementary Fig. 1. Sensitivity of the target amplification and SHARD PCRs.

A: Representative gel picture of target amplification PCR products using wild type and mutant pJET-promoter vectors as templates. B: Chart summarizing the five distinct experiments, which through we explored the amplification threshold of the wild type and two mutant promoters

(mean with standard error of mean (SEM)). This way the five independent experiment tested the promoter amplification 15 times and resulted 0.454 pg of template mass as amplification threshold. Interestingly, despite a mere one base pair distinction, the mutant promoters seem to exhibit a lower amplification threshold (0.442, 0.28) compared to the wild type (0.64), although this variance is not statistically significant. C: Representative gel picture shows SHARD-PCR products that were amplified from templates of mixed wild-mutant fragments ranging from 50% to 0.005% ratio. Asterisk shows aspecific signal, which was observed when using older batch of DNA oligomers. D: The results from 5-5 SHARD-PCR sensitivity experiments indicate that the mutant ratio must exceed 1.3% for the -124M case and 2.3% for the -146M case, as shown by the mean with SEM values on the chart.

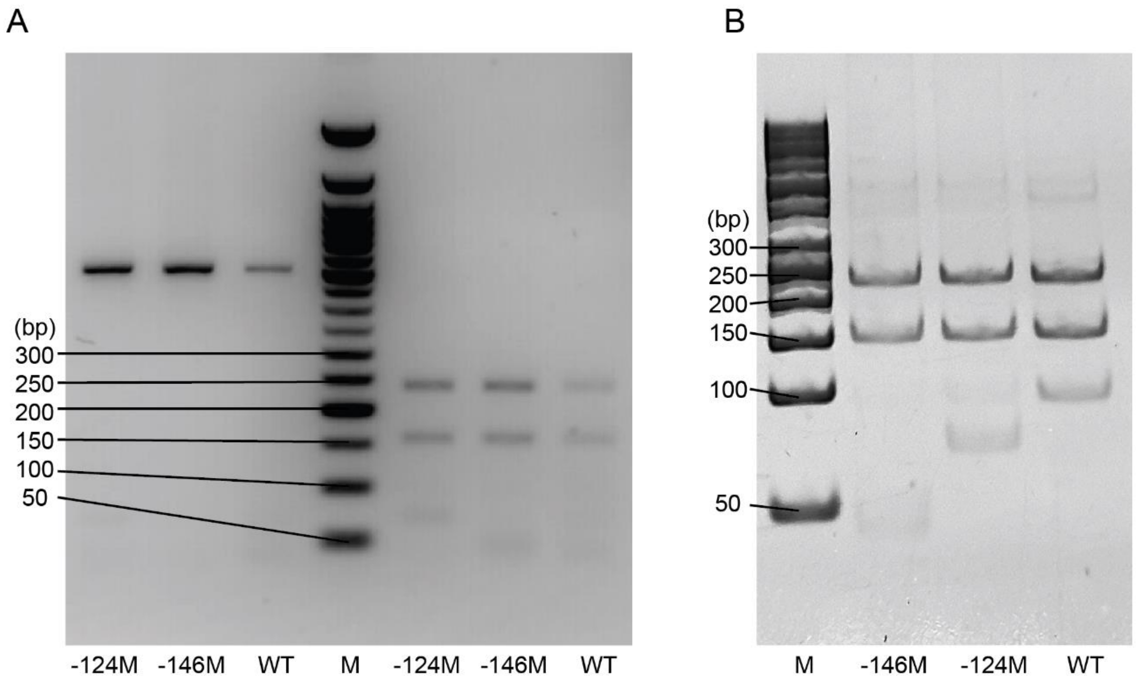

Supplementary Fig. 2. A: First-step PCR products of mutant cell line DNA before and after HpyAV restriction digestion (3% agarose gel). B: First-step PCR products of mutant cell line DNA after HpyAV restriction digestion (8% acrylamide gel).

A

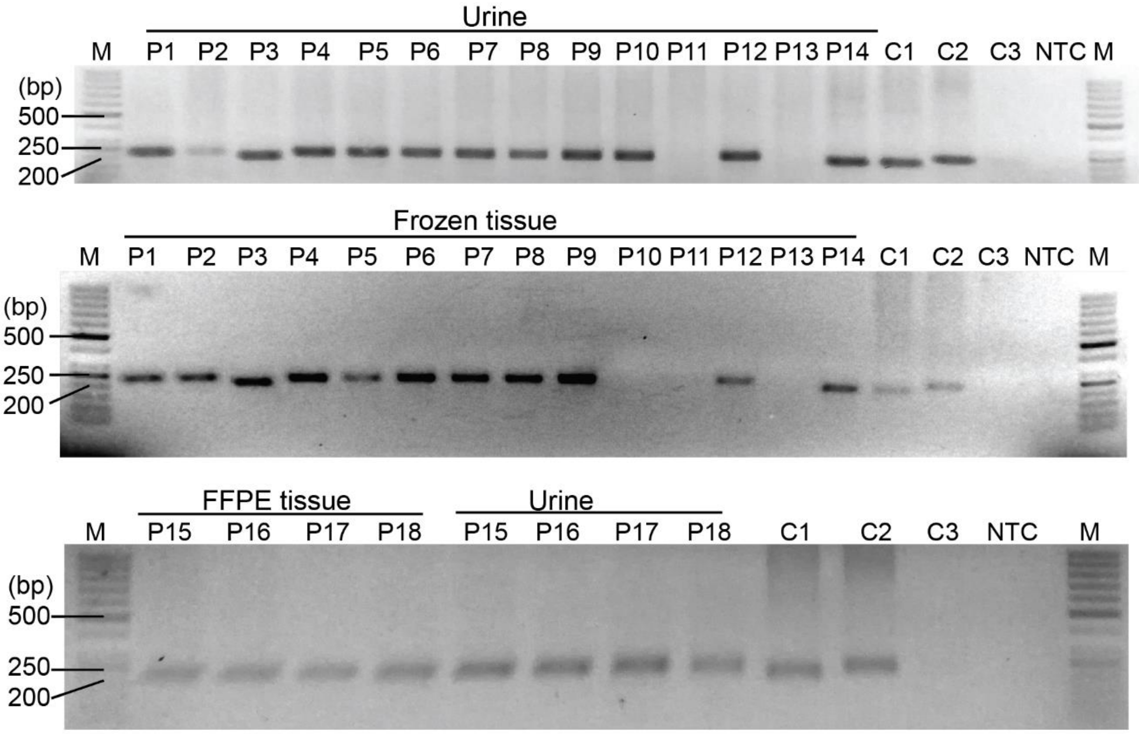

B

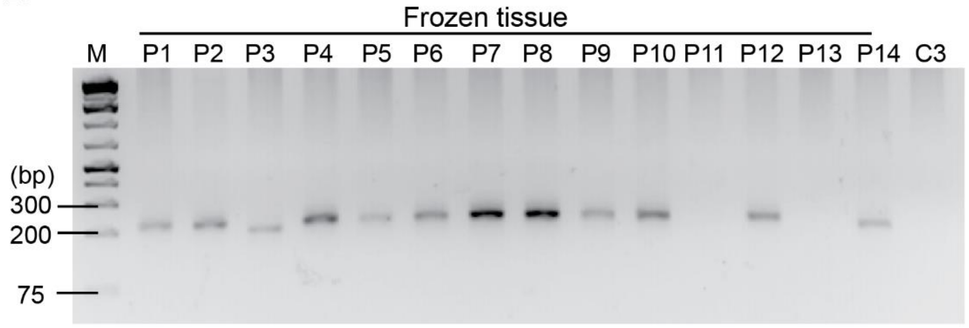

Supplementary Fig. 3. Uncropped agarose gel images presented in this study. A: Urine and tissue samples examined with the “Q5 protocol”. B: Tissue samples examined with the “DreamTaq protocol”.

Supplementary Table 1. The sequences of primers used were:

**489 Forward Primer**	**5’GGCCGATTCGACCTCTCT3’**
**489 Reverse Primer**	5’AGCACCTCGCGGTAGTGG3’
**Blocked Oligo A146**	5’CGGAGCACGTGCGACCCAGTTCAGCGTACTAGTCATCGTAGCGTCGTGCTAGCATTCATGACCCGGAAGGGGTCGGGACGTddC3’
**Blocked Oligo A124**	5’CGGAGCACGTGCGACCCAGTTCAGCTCATGCAACTTAAGGTCTGAGGTCGCTAAGCGATCTGGCCCGGAAGGGGCTGGGCCGTddC3’
**Extension Primer 1**	5’GGTAGTTATGTTTGTTGTTTTAGTATAGTTTGTTGTTTGTTTATTTGTTTCGGAGCACGTGCGACCCAGTTCAGC3’
**Extension Primer 2**	5’GGTAGTTATGTTTGTTGTTTTAGTATAGTTTGTTGTTTGTTTATTTGTTTCGCCTCCTCCGCGCGGACCC3’
**Forward-Reverse Primer**	5’GGTAGTTATGTTTGTTGTTTTAGTATAGTTTGTTGTTTGTTTATTTGTTTCG3’
**Blocked Oligo B146**	5’CGGAGCACGTGCGACCCAGTTCAGCGTACTAGTCATCGTAGCGTCGTGCTAGCATTCATGACCCGGAAGGGGTCGTTTAA3’
**Blocked Oligo B124**	5’CGGAGCACGTGCGACCCAGTTCAGCTCATGCAACTTAATGAGGTCGCTAAGCGATCTGGCCCGGAAGGGGCTGAATTA3’
**Sequencing Primer 1**	5’ACACTCTTTCCCTACACGACGCTCTTCCGATCTCTGCCTGAAACTCGCGCCG3’
**Sequencing Primer 2**	5’ATCTCGTATGCCGTCTTCTGCTTGGGCCGATTCGACCTCTCT3’

Supplementary Table 2. Mutations identified by NGS and PCR

Code	Sex	Age (years)	Tumor Grade	pT	Mutation by NGS - Tumor tissue (Type - %)	Mutation by the method - Tumor tissue	Mutation by NGS - Urine (Type - %)	Mutation by the method - Urine
P1	F	74	High grade	pTx	-124M - 82%	-124M	-124M - 65%	-124M
P2	M	66	High grade	pT1	-124M - 84%	-124M	-124M - 55%	-124M
P3	M	64	High grade	pT2a	-146M - 68%	-146M	-146M - 68%	-146M
P4	M	58	High grade	pT2b	-124M - 85%	-124M	-124M - 60%	-124M
P5	M	67	Low grade	pTx	-124M - 76%	-124M	-124M - 46%	-124M
P6	M	65	High grade	pT2	-124M - 80%	-124M	-124M - 20%	-124M
P7	M	73	High grade	pT2	-124M - 78%	-124M	-124M - 76%	-124M
P8	M	40	Low grade	pTx	-124M - 75%	-124M	-124M - 91%	-124M
P9	M	66	High grade	pT3b	-124M - 56%	-124M	-124M - 62%	-124M
P10	M	51	High grade	pT2a	-124M - 43%	-124M	-124M - 76%	-124M
P11	M	70	High grade	pTx	WT - 100%	WT	WT - 97%	WT
P12	M	63	High grade	pT2	-124M - 45%	-124M	-124M - 74%	-124M
P13	F	63	High grade	pT2	WT - 100%	WT	WT - 100%	WT
P14	M	64	High grade	pT1	-146M - 68%	-146M	-146M - 61%	-146M
P15	M	63	High grade	pTa	-124M - 78%	-124M	-124M - 69%	-124M
P16	M	83	High grade	pTx	-124M - 93%	-124M	-124M - 53%	-124M
P17	M	84	High grade	pTx	-124M - 89%	-124M	-124M - 48%	-124M
P18	M	68	Low grade	pTx	-124M - 90%	-124M	-124M - 8%	-124M
